# Evaluation of a glycoengineered monoclonal antibody via LC-MS analysis in combination with multiple enzymatic digestion

**DOI:** 10.1080/19420862.2015.1113361

**Published:** 2015-10-29

**Authors:** Renpeng Liu, John Giddens, Colleen M. McClung, Paula E. Magnelli, Lai-Xi Wang, Ellen P. Guthrie

**Affiliations:** a New England Biolabs Inc., Ipswich, MA 01938; b Department of Chemistry & Biochemistry; University of Maryland, College Park, MD 20742

**Keywords:** antibody characterization, glycoengineering, glycosylation analysis, LC-MS, procainamide

## Abstract

Glycosylation affects the efficacy, safety and pharmacokinetics/pharmacodynamics properties of therapeutic monoclonal antibodies (mAbs), and glycoengineering is now being used to produce mAbs with improved efficacy. In this work, a glycoengineered version of rituximab was produced by chemoenzymatic modification to generate human-like *N*-glycosylation with α 2,6 linked sialic acid. This modified rituximab was comprehensively characterized by liquid chromatography-mass spectrometry and compared to commercially available rituximab. As anticipated, the majority of *N*-glycans were converted to α 2,6 linked sialic acid, in contrast to CHO-produced rituximab, which only contains α 2,3 linked sialic acid. Typical posttranslational modifications, such as pyro-glutamic acid formation at the N-terminus, oxidation at methionine, deamidation at asparagine, and disulfide linkages were also characterized in both the commercial and glycoengineered mAbs using multiple enzymatic digestion and mass spectrometric analysis. The comparative study reveals that the glycoengineering approach does not cause any additional posttranslational modifications in the antibody except the specific transformation of the glycoforms, demonstrating the mildness and efficiency of the chemoenzymatic approach for glycoengineering of therapeutic antibodies.

## Abbreviations

LC-MS: liquid chromatography-mass spectrometry
mAbs: monoclonal antibodies
ADCC: antibody-dependent cell-mediated cytotoxicity
CDC: complement-dependent cytotoxicity
CHO: Chinese hamster ovary
α-Gal: alpha linked galactose
Neu5Gc: *N*-glycolylneuraminic acid
Neu5Ac: *N*-acetylneuraminic acid
HILIC: hydrophilic interaction liquid chromatography
2-AB: 2-aminobenzamide
PCA: procainamide
pyro-E: pyro-glutamic acid
GuHCl: guanidinium hydrochloride
DTT: dithiothreitol
IAM: iodoacetamide.

## Introduction

Monoclonal antibodies (mAbs) have become increasingly important as innovative therapeutic agents during the past 2 decades. Consequently, the biopharmaceutical industry is now dedicating extensive efforts to developing new types of antibody-based therapeutics, including bispecific antibodies, Fc-fusion proteins and antibody-drug conjugates.^1^ At the same time, biosimilar and biobetter products have attracted tremendous interest due to the upcoming expiration of patent protection for many commercially successful mAbs.^2^ Therefore, substantial efforts have also been dedicated to exploring safer and more efficacious products that improve upon the currently marketed mAbs. In particular, one area of research has focused on improving antibody properties by controlling the composition of glycosylation.^3-6^ The antibody Fc domain bears 2 *N*-glycans at the conserved *N*-glycosylation sites (N297 in IgG1), and the *N*-glycan profile of therapeutic antibodies has an important effect on protein stability, structural stability and clinical efficacy.^7-8^ For instance, glycosylation has been shown to be critical for antibody-dependent cell-meditated cytotoxicity (ADCC) and complement-dependent cytotoxicity (CDC). Approaches to develop glycoengineered mAbs with improved efficacy include *N*-glycosylation humanization and alternative cell line expression to obtain a desired glycoform, which yielded low fucose-content mAbs that demonstrated enhanced ADCC in cancer treatment.^3^ The approval of obinutuzumab (Gazyva®), a glycoengineered anti-CD20 mAb with enhanced ADCC, underscored the importance and the viability of glycoengineering approach.^9^

Most marketed therapeutic mAbs are produced either in Chinese hamster ovary (CHO) or mouse myeloma (NS0, SP2/0) cell lines. However, it was found that the glycosylation of protein drugs produced in these cells is significantly different than that produced in human cells, and this raises concerns regarding immunogenicity of the drugs.^5,10-12^ For example, α-linked galactose (α-Gal) and *N*-glycolylneuraminic acid (Neu5Gc) structures are found in murine IgG glycans, while only *N*-acetylneuraminic acid (Neu5Ac) is present in human glycoproteins. Both α-Gal and Neu5Gc residues have been found to be immunogenic.^10,13,14^ CHO cells do not express α-Gal, but the number of terminal β-galactose residues (generally referred to as G0, G1 and G2) of mAbs produced in these cells is variable.

Because of protein quality and consistency requirements, controlling glycan heterogeneity is desirable and essential. One approach is to enhance recombinant protein sialylation and thus prevent protein degradation and increase the in vivo circulatory half-life of the proteins. The α2,6-linkage of sialic acid is the most common terminal glycosylation of human proteins, and it is widely regarded as the representative human-type glycosylation.^15-18^ However, CHO cells are unable to produce α2,6 linked sialic acid. Terminal α2,6-sialylation could also be important for antibody efficacy.^19^ It has been found that terminal α2,6-sialylation of Fc glycans plays a critical role for the anti-inflammatory activity of human intravenous immunoglobulins, while terminal α2,3-sialylation did not have anti-inflammatory activity.^20^ This discovery underscores the importance of α2,6-sialylation on the antibody glycans. Indeed, numerous approaches are currently under investigation to generate α2,6-linked sialic acids to produce glycoproteins with human-like glycoform patterns. These methods include expression of α2,6-linkage specific sialyltransferase ST6Gal-1 (ST6) activity in the CHO cells and *N*-glycan remodeling using ST6 in vitro. More recently, a new approach to co-express ST6 and β1,4-galactosyltransferase 1 (GT) in CHO cell yielded antibody on which 85% of the sialic acids were α2,6-linked.^21^ However, this approach still couldn't achieve the goal of well-defined homogeneous glycoforms with nearly 100% of α2,6-linked sialic acids.

**Figure 1.**
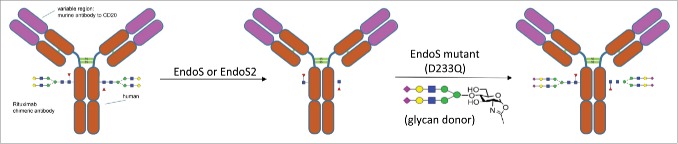
Glycosylation remodeling of rituximab to prepare rituximab with homogenous *N*-glycan with 2,6 linked sialic acid using EndoS or EndoS2.

Recently, a novel glycoengineering approach for intact IgG glycosylation modification was developed.^22-25^ As demonstrated in a previous model study,^23^ by taking advantage of endoglycosidase Endo S and Endo S mutants (D233A and D233Q), rituximab, an anti-CD20 mAb, was successfully transformed from a mixture of G0F, G1F and G2F glycoforms to well-defined homogeneous glycoforms (**Fig. 1**). These included a fully sialylated (G2S2F) glycoform that may gain anti-inflammatory activity, as well as a non-fucosylated G2 glycoform that showed significantly enhanced FcγIIIa receptor binding activity, therefore enhancing ADCC.

In most previous model studies involving glycoengineering of antibodies, the characterization was mainly focused on the glycan portions without detailed characterization of the protein portions. However, it is important to also clarify whether the glycoengineering processes would cause any other posttranslational modifications of the mAbs. A recent report indicated that some enzymatic glycosylation processes could led to unexpected modifications on the protein portions of antibodies, probably due to contaminations of the enzymes.^26^ Indeed, other common chemical modifications such as oxidation, deamidation, N-terminal pyroglutamic acid cyclization and C-terminal lysine removal are also important for antibody quality and efficacy.^2^ In this study, we developed multiple enzymatic digestion, combined with a novel fluorescent tag and state of the art mass spectrometric methods to fully characterize the glycan structure of the newly developed fully sialylated (G2S2F) mAb with α2,6-sialylation, and compared it to the structure of the commercial rituximab molecule. The primary structure, disulfide linkage, and common modifications, such as pyro-glutamic acid formation at the N-terminus, oxidation at methionine, deamidation at asparagine, and C-terminal lysine processing were also investigated. Our study reveals that this glycoengineering process does not cause any posttranslational modifications except the specific transformation of the antibody glycoforms.

## Results

### Analysis of glycosylation

Due to the important role of glycosylation for the effector function of mAbs, many analytical techniques are used to determine the *N*-glycan profile. Liquid chromatography-mass spectrometry (LC-MS) analysis is a commonly employed technique because of its sensitivity and ability to quantitate when labeled glycans are used. When using LC-MS, it is important to choose an appropriate type of chromatography for efficient LC separation. One common method, reverse phase LC-MS, is unable to differentiate glycans with α2,3-linkage or α2,6-linkage terminal sialic acid. Therefore, the glycans were released using PNGase F and analyzed by hydrophilic interaction liquid chromatography (HILIC)-MS.

Since they lack a chromophore, most glycans are difficult to detect when analyzed by high performance (HP)LC, and it is common practice to form a derivative at the reducing end to enable detection by fluorescence and mass spectrometry. While 2-aminobenzamide (2-AB) is the most widely used fluorescent tag, poor glycan ionization efficiency limits its identification and characterization of minor glycan species. Instead, a fluorescent tag, procainamide (PCA) was used for analysis of *N*-glycans from rituximab because the procainamide derivatives have been reported to produce fluorescent glycan profiles comparable to the 2-AB derivatives with much improved ionization efficiency.^27,28^

Using fluorescent detection with LC-MS, we identified a total of 24 *N*-glycans from commercial rituximab, including G0F, G1F and G2F structures as the major forms, and the non-fucosylated complex and high–mannose type *N*-glycans as the minor forms (**Table 1**). The number of *N*-glycans identified was higher than what was previously reported,^2^ mainly due to the better ionization efficiency of procainamide derivatives. We also characterized *N*-glycans from the glycoengineered mAb. As we expected, the majority of the peaks are G2S2F, with a small amount of the glycan being the afucosylated G2S2 form. We also observed high-mannose-type glycans, such as the Man5 structure that was not changed before and after the Endo S-based glycoengineering process. This is due to the known substrate specificity of Endo S, which prefers complex type *N*-glycans at the Fc domain as the substrate and shows poor hydrolytic activity toward high-mannose type Fc *N*-glycans.^29^ Because of this limitation, the Man5 glycan could not be converted into the G2S2 structure through this approach. However, this problem could be overcome by using a recently discovered enzyme, Endo S2, which can cleave a broader range of *N*-glycans including high-mannose type structures.^29^

**Table 1.** Summary of rituximab *N*-glycan.

Glycan	Retention time (min)	m/z	Peak area %^*^
Man3	8.88	1030.69 (1+)	0.10%
Man3F	9.37	1276.61 (1+)	0.14%
G0-N	10.53	1333.78 (1+)	0.17%
G0F-N	12.36	1479.87 (1+)	0.99%
G0	12.84	1536.87 (1+)	0.78%
G0F	14.83	1683.05 (1+)	36.44%
Man5	16.51	1454.87 (1+)	1.68%
G1F-N	17.26	1641.96 (1+)	1.26%
G1a	17.26	1698.96 (1+)	0.36%
G1b	17.70	1698.96 (1+)	0.16%
G1Fa	19.35	1845.05 (1+)	30.60%
G1Fb	20.09	1845.05 (1+)	9.83%
Man6	21.63	1616.88 (1+)	0.51%
G1FS1-N	22.04	1933.14 (1+)	1.00%
G1FS1a	23.58	1068.61 (2+)	0.65%
G1FS1b	24.80	1068.61 (2+)	0.11%
G2F	25.34	1004.11 (2+)	9.42%
Man7	27.64	1779.05 (1+)	0.98%
G2FS1a	29.78	1149.61 (2+)	1.34%
G2FS1b	30.34	1149.61 (2+)	0.78%
M5N3G1FS1	31.14	1129.11 (2+)	0.37%
M5N4G1F	31.14	1085.11 (2+)	0.24%
Man8	33.45	1941.14 (1+)	0.69%
G2FS2	35.06	1295.20 (2+)	1.38%

* MS-signal ratios used for co-eluting fluorescent signal

**Figure 2.**
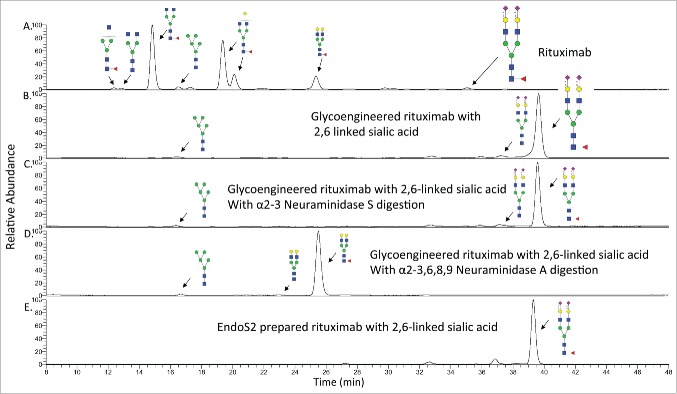
HPLC-FLD profile of procainamide labeled *N*-glycans from rituximab and the glycoengineered rituximab with α2,6 linked sialic acid. A). HPLC profile of rituximab *N*-glycans with major species shown. B). The glycan profile from the glycoengineered rituximab prepared by Endo S digestion. The major glycan is G2FS2 with α2,6 linked sialic acid. C). The glycan profile from the glycoengineered rituximab after α2-3 Neuraminidase S treatment. D). The glycan profile from the glycoengineered rituximab after α2-3,6,8,9 Neuraminidase A treatment. E). The glycan profile from the glycoengineered rituximab prepared by Endo S2 digestion.

The preparation of a glycoengineered rituximab was repeated by using Endo S2 treatment, leading to the complete removal of *N*-glycans. The different elution time of G2S2F structures (35.06 min vs 39.65 min) clearly indicated the linkage was converted from α2,3-linked to α2,6-linked sialic acid (**Fig. 2A and B**). This is consistent with the well-known observation that glycans containing α2,6-linked sialic acid would elute later than similar glycans with α2,3-linked sialic acid. The linkage was further confirmed by digestion with 2 different neuraminidases. When the glycoengineered antibody was treated with α2-3 Neuraminidase S, an enzyme that can only remove α2,3-linked sialic acid, no new products were observed in the LC trace, indicating that α2-3 sialic acid was not present (**Fig. 2C**). However, when the glycoengineered antibody was treated with the α2-3,6,8,9 Neuraminidase A, an enzyme with broad specificity that is able to remove sialic acid residues regardless of linkage, a shift in the LC trace was observed, indicating the presence of α2,6-linked sialic acids (**Fig. 2D**). The Man5 structure was also observed in the Endo S prepared sample (**Fig. 2B**), whereas the glycoengineered rituximab prepared by Endo S2 treatment showed no Man5 structures (**Fig. 2E**). These results show that the *N*-glycans from rituximab are indeed transformed from G0F, G1F, G2F, Man5 and other related glycoform structures into mainly S2G2F with α2,6-linked sialic acid in the glycoengineered antibody.

### Disulfide linkage analysis

Correct disulfide bond formation is important for mAb structure and function. Enzymatic digestion without reduction followed by LC-MS was used for disulfide bond analysis. All the expected disulfide linkages were identified by accurate precursor mass measurement and collision-induced dissociation fragmentation of the precursor ion.^2,30,31^ Rituximab has 8 disulfide bonds, 6 of which could be found in the trypsin digestion product. The other 2 were not identified from the trypsin digestion, but could be identified using Lys-C digestion (**Fig. S1; Table S1**). Therefore, using a combination of Lys-C and trypsin digestion, all 8 expected disulfide bonds were found and confirmed in both commercial and glycoengineered mAbs.

### Chemical modifications

MAbs can undergo numerous modifications, including deamidation and oxidation, during the manufacturing, formulation, purification and storage.^32^ It is known that these modifications will affect the stability and efficacy of mAbs. Non-enzymatic deamidation of asparagine (Asn) is a common modification. The most susceptible sites for deamidation occur where Asn is followed by Gly, Ser or Thr (Asn-Gly, Asn-Ser or Asn-Thr). The first step of the process is a loss of amine in the side chain of asparagine to form a succinimide intermediate, and this intermediate is eventually hydrolyzed to aspartic acid or isoaspartic acid. Compared to asparagine residues, the mass of the succinimide intermediate is 17 Da less, while the mass of the complete deamidation product, aspartic or isoaspartic acid, is 1 Da more.^33^

**Figure 3.**
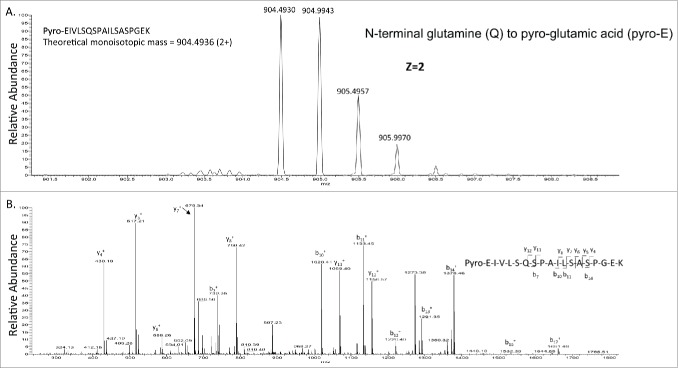
The detection of pyro-E in rituximab. A). Precursor mass of pyro-glutamic acid of the heavy chain from rituximab. B). CID-MS^2^ of the precursor mass from **Figure 3A**. The theoretical and observed monoisotopic mass are indicated in the figure.

Enzymatic peptide mapping using trypsin digestion is widely used to map mAbs. In order to reduce the deamidation artifact during the trypsin digestion in ammonium bicarbonate, a fast digestion protocol (35 mins) adapted from a previous publication^34^ was used. Only succinimide intermediates were observed. Oxidation of methionine to methionine sulfoxide is a hotspot for oxidation, and this product was also found in rituximab in this study. An additional modification often found in mAbs is the conversion of “glutamine (Q)” or “glutamic acid (E)” N-terminal amino acids to “pyro-glutamic acid (pyro-E),” although this modification may not affect the efficacy of mAbs.^2,35^ The N-terminus of heavy and light chains of rituximab contain the glutamate amino acid; therefore, the occurrence of pyro-E peptides within N-terminal peptides (minus 17 Da due to the loss of amine) was also explored (**Fig. 3**). Removal of the heavy chain C-terminal lysine by carboxypeptidases during cell culture production is also one of the most common modifications of mAbs. Similar to N-terminal pyro-E cyclization, C-terminal lysine processing is easily detected by MS and has no significant influence on antibody structure or function. Percentage of succinimide intermediate, methionine oxidation, C-terminal lysine removal and N-terminal pyro-E were calculated by using the peak areas from extracted ion chromatography of modified and unmodified peptides (**Figs. S2–7**). The summary of chemical modifications identified is in **Table S2**. No significant differences were observed for these modifications for the commercially available and the glycoengineered mAbs, indicating that the in vitro enzymatic *N*-glycosylation modification approach used in this paper does not introduce significant changes in these important posttranslational modifications.

## Discussion

All characteristics of commercially available and glycoengineered mAb, including peptide sequence, glycosylation, correct disulfide bonds, and common modifications, were studied and identified by LC-MS based analysis. The use of procainamide labeling combined with LC-MS identified more glycans in the commercially available rituximab than previously reported, indicating the power of using this new labeling technique. Also, 2 neuraminidases were used to identify the linkage of sialic acid. The combination of procainamide labeling, LC-MS and exo-glycosidase sequencing greatly improves the identification of glycan structures in mAbs. Using the Endo S-based glycoengineering procedure, the glycans have been mainly converted to G2S2F or G2S2 structures, while the Man5 structure was kept intact during the engineering process due to the limitation of Endo S substrate specificity. This problem could be overcome by using Endo S2 as the enzyme for deglycosylation, which can cleave high-mannose structures. Therefore, Endo S2 treatment produces an antibody with more homogenous glycans. The glycosylation can be further optimized by removing core-fucose through treatment with a fucosidase to enhance ADCC.

Unlike previous studies in which only glycosylation was compared between commercially available and glycoengineered products, the present study investigated common chemical modifications such as N-terminal pyroglutamic acid cyclization, C-terminal lysine removal, oxidation, and deamidation, together with glycosylation in both commercially available and glycoengineered product. Similar levels of modifications were observed by using a relative quantitation approach. These results indicate that the in vitro glycosylation engineering approach used in this work does not introduce significant changes in these important posttranslational modifications and could be a powerful approach to develop biobetters with homogenous humanized glycosylation.

## Materials and methods

### Materials

The therapeutic mAb rituximab (Rituxan®, Genentech, Inc.) was purchased through Premium Health Services Inc. (NDC code# 50242-0051-21). Sialoglycan oxazoline was synthesized following a previously reported procedure.^23^ Trypsin-ultra (P8101S), PNGase F (P0708S), α2-3 Neuraminidase S (P0743S), α2-3,6,8,9 Neuraminidase A (P0722S), Remove-iT Endo S (P0741S) are products from New England Biolabs. Endo S2 was purchased from Genovis (A0-GL1-020). Lys-C was purchased from Wako Chemicals USA, Inc. (125-05061). Ammonium bicarbonate (09830), guanidinium hydrochloride (GuHCl) (G4505), dithiothreitol (DTT) (43819), iodoacetamide (IAM) (I6125), Tris base (T1503) and procainamide (4-amino-*N*-(2-diethylaminoethyl)benzamide) (P9391) were obtained from Sigma-Aldrich. LC-MS grade water (BJLC365-1) and acetonitrile (34967) were purchased from VWR. Amicon centrifugal filters (100 kDa and 10 kDa molecular weight cutoff) were obtained from EMD Millipore (UFC501096 and UFC510024 respectively). NAP-5 columns (17-0853-02) were obtained from GE Healthcare. HILIC Microspin column (SEM-HIL) was purchased from The Nest Group, Inc.

### Preparation of rituximab with α2,6-linked sialic acid

Wild type and mutant Endo S were expressed following a previous procedure,^23^ and the rituximab with α2,6-linked sialic acid was prepared following a previous protocol.^23^. Briefly, commercial rituximab in a Tris-Cl buffer (100 mM, pH 7.4, 2 mL) was incubated with Endo S at 37°C for 1 h. LC-MS and SDS-PAGE analysis indicated the complete cleavage of the *N*-glycans on the heavy chain. The reaction mixture was subject to affinity chromatography on a column of protein A-agarose resin (1 ml) that was pre-equilibrated with a Tris-Cl buffer (20 mM, pH 8.0). The column was washed with Tris-Cl (20 mM, pH 8.0, 25 ml) and glycine-HCl (20 mM, pH 5.0, 20 ml) successively. The bound IgG was released with glycine-HCl (100 mM, pH 2.5) and the elution fractions were immediately neutralized with Tris-HCl buffer (1.0 M, pH 8.8). The fractions containing antibodies were combined and concentrated by centrifugal filtration (Amicon® Ultra centrifugal filter, to give (Fucα1,6)GlcNAc-rituximab. A solution of (Fucα1,6)GlcNAc-rituximab and α2,6 sialic acid oxazoline in a Tris buffer (100 mM, pH 7.4, 0.5 mL) was then incubated with Endo S-D233Q (200 μg) at 37°C. Aliquots were taken at intervals and were analyzed by LC-MS. After 2 h, LC-MS monitoring production of the transglycosylation product was found to be complete. The reaction mixture was subject to affinity chromatography on a column of protein A as described above. Fractions containing the product were combined and concentrated by ultracentrifugation to give the rituximab with α2,6-linked sialic acid glycoform. The rituximab treated by Endo S2 was produced by the same procedure as the Endo S-treated rituximab.

### Enzymatic digestion

To reduce trypsin digestion artifacts, a protocol for fast digestion was adapted from a previous report.^34^ Briefly, an aliquot of 500 μL of rituximab and glycoengineered mAb solution (100 μg) was denatured with 7.5 M guanidine hydrochloride containing 50 mM Tris (pH 7.0), reduced with 8 mM DTT for 30 min at 37°C, and then alkylated with 14 mM IAM in the dark for 30 min at room temperature. Excess IAM was neutralized by 6 mM DTT. The reduced and alkylated protein was buffer exchanged with 100 mM ammonium bicarbonate (pH 8) using a NAP-5 column. For tryptic digestion, trypsin (1:10, w/w) was added to the protein solution at 37°C for 35 mins. For Lys-C digestion, the Lys-C (1:20, w/w) was added to protein solution for 16 hours at 37°C. For glycan analysis, the Fc-glycans were released using PNGase F. The *N*-glycans were then labeled with procainamide using a standard protocol.^28^ The labeled glycan were purified by HILIC-SPE using a HILIC Microspin column.

### LC-MS/MS for peptide analysis and glycosylation analysis

A Proxeon Easy nano-LC 1000 pump (Thermo Fisher Scientific) was coupled online to an LTQ-Orbitrap-ETD XL mass spectrometer (Thermo Fisher Scientific) through a nanospray ion source (New Objective). Mobile phase A (0.1% formic acid in water) and mobile phase B (0.1% formic acid in acetonitrile) were used for the gradient consisting of: 1) 2 min at 5% B; 2) linear from 5 to 40% B for 60 min; 3) linear from 40 to 100% B for 7 min; and finally 4) isocratic at 100% B for 5 min. The flow rate of the column was maintained at 0.5 μL/ min. The LTQ-Orbitrap-ETD XL mass spectrometer was operated initially in data-dependent mode as follows: survey full-scan MS spectra (m/z 400-1600) were acquired in the Orbitrap with a mass resolution of 30,000 at m/z 400, followed by 5 sequential MS^2^ scans using the LTQ. An aliquot of 18 μL (3 μg) of the enzyme digest was analyzed per LC-MS run. Each sample was repeated 3 times. For glycosylation analysis, a Dionex 3000 UHPLC (Thermo Fisher Scientific) was coupled online to a LTQ Velos Pro mass spectrometer (Thermo Fisher Scientific). For HPLC separation, a Waters Amide XP column (2.1 mm × 150 mm, 2.5 µm particle size) was used for analysis. Fluorescence at 308 nm excitation and 359 nm emission were used to detect procainamide-glycan conjugates in HPLC analyses and separations. Mobile phase A (50 mM ammonium formate, pH 4.4) and mobile phase B (100% acetonitrile) were used for the gradient consisting of: 1) 2 min at 69% B; 2) linear from 69 to 65% B for 24 min; and 3) linear from 65 to 57% B for 24 min. The flow rate of the column was maintained at 0.3 mL/ min and column temperature was maintained at 30°C.

### Peptide and disulfide assignment

MS/MS data were analyzed manually using Xcalibur software for mass detection and data interpretation. Peptides of interest were identified manually by searching their m/z-values within the experimental mass spectrum. Relative amounts of common chemical modifications were calculated by manual integration of modified and unmodified peptide peaks.

## Supplementary Material

Liu et al Supplemental Data
