# Supplementary material for: Evaluation of a glycoengineered monoclonal antibody via LC-MS analysis in combination with multiple enzymatic digestion
Source: MAbs. 2015 Oct 29;8(2):340–6. doi: 10.1080/19420862.2015.1113361 (PMC4966608; doi:10.1080/19420862.2015.1113361)
Supplement: Liu et al Supplemental Data [file kmab-08-02-1113361-s001.zip › SuppTable2.pdf]

Supplementary Table 2: comparison of common chemical modifications in rituximab innovator and glycoengineered mAb.

	Innovator	Glycoengineered	P value
Deaminidation in succinimide form (n=3)			
N55	1.5±0.1	1.7±0.1	0.134
N319	1.4±0.1	1.6±0.2	0.261
N388	1.0±0.2	1.3±0.0	0.122
Oxidation (n=3)			
Met34	10.1±2.7	11.1±2.5	0.685
Met256	7.8±0.6	9.9±0.9	0.078
N-terminal and C-terminal modifications (n=3)			
Pyro-glu at heavy chain	100±0.0	100±0.0	n.a.
Pyro-glu at light chain	100±0.0	100±0.0	n.a.
K-clipping at C-terminal	99.1±0.1	99.1±0.1	n.a.

Extracted ion chromatography (XIC) was used to calculate %. $P \leq 0.05$ for statistical significance
