# Supplementary material for: Evaluation of a glycoengineered monoclonal antibody via LC-MS analysis in combination with multiple enzymatic digestion
Source: MAbs. 2015 Oct 29;8(2):340–6. doi: 10.1080/19420862.2015.1113361 (PMC4966608; doi:10.1080/19420862.2015.1113361)
Supplement: Liu et al Supplemental Data [file kmab-08-02-1113361-s001.zip › SuppTable1.pdf]

Supplementary Table 1: Summary of disulfide bond identification in rituximab.

Disulfide linkage sequence	Enzyme	Retention time (min)	Theoretical monoisotopic mass
MSCK<br>SSSTAYMQLSSLTSEDSAVYYCAR	Trypsin	37.53	3084.318
DYFPEPVTVSWNSGALTSGVHTFPAVLQSSG LYSLSSVVTVPSSSLGTQTYICNVNHKPSNTK<br>STSGGTAALGCLVK	Trypsin	50.39	7916.919
SCDK<br>SFNRGEC	Lys-C	9.73	1260.486
THTCPPCPAPELLGGPSVFLFPPK<br>THTCPPCPAPELLGGPSVFLFPPK	Lys-C	49.36	5004.487
CK<br>TPEVTCVVVDVSHEDPEVK	Trypsin	30.31	2328.097
NQVSLTCLVK<br>WQQGNVFSCSVMHEALHNHYTQK	Trypsin	35.48	3844.823
VTMTCR<br>VEAEDAATYYCQQWTSNPPTFGGGTK	Trypsin	37.06	3527.543
SGTASVVCLLNNFYPR<br>VYACEVTHQGLSSPVTK	Trypsin	40.26	3555.748
