# Supplementary figures and images for: Evaluation of a glycoengineered monoclonal antibody via LC-MS analysis in combination with multiple enzymatic digestion
Source: MAbs. 2015 Oct 29;8(2):340–6. doi: 10.1080/19420862.2015.1113361 (PMC4966608; doi:10.1080/19420862.2015.1113361)

# Supplementary Figure 1

A.

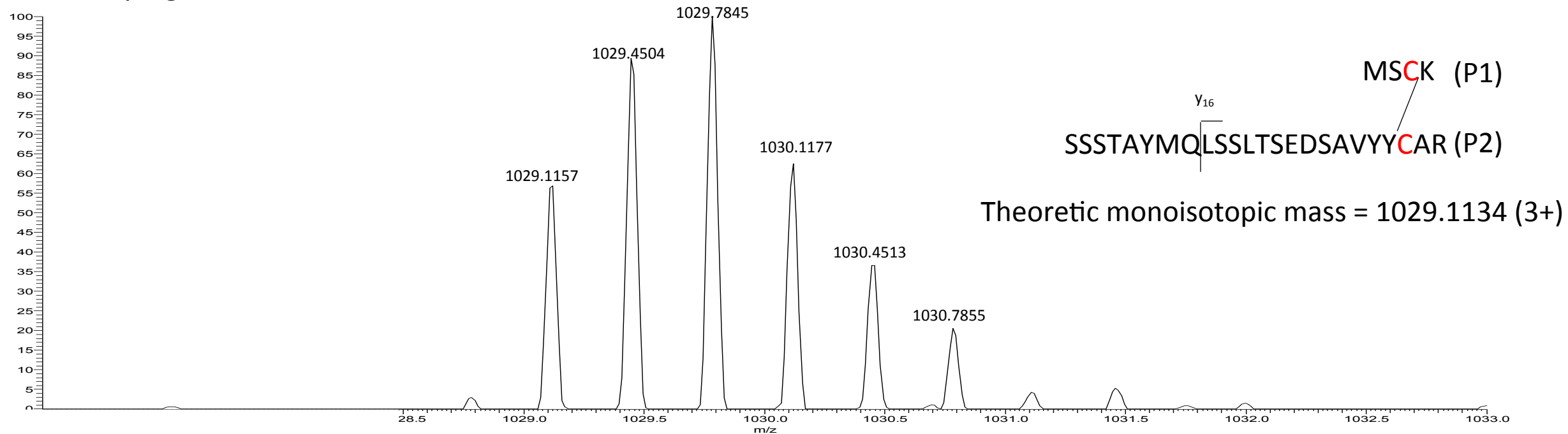

B.

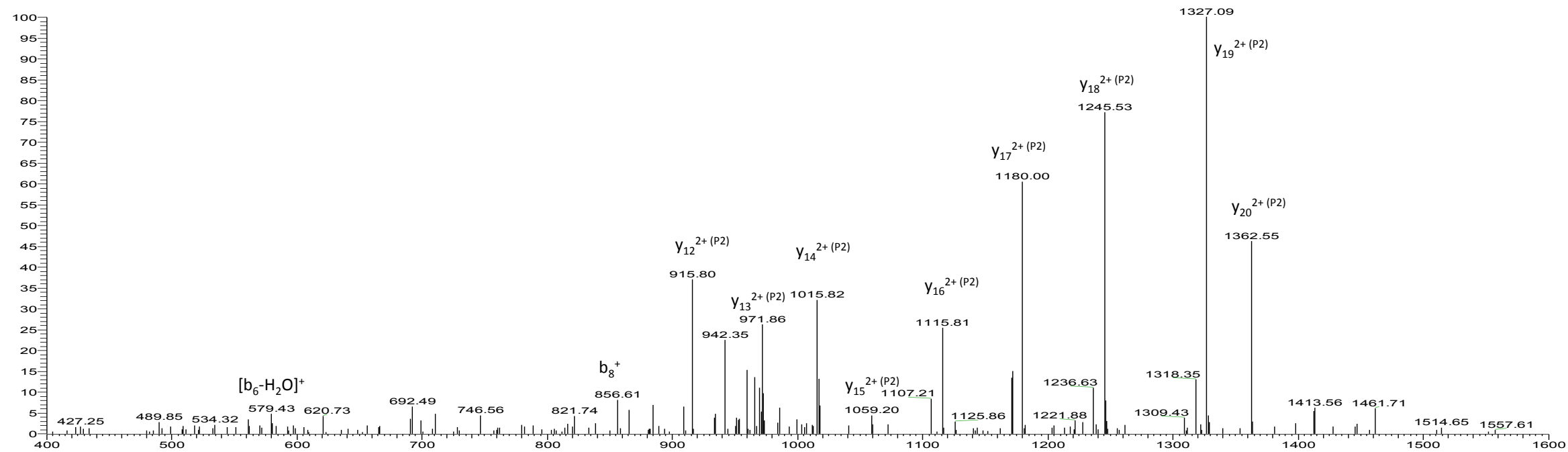

Supplement: Liu et al Supplemental Data [file kmab-08-02-1113361-s001.zip › SuppFigure1.pdf]

Supplementary Figure 2

A.

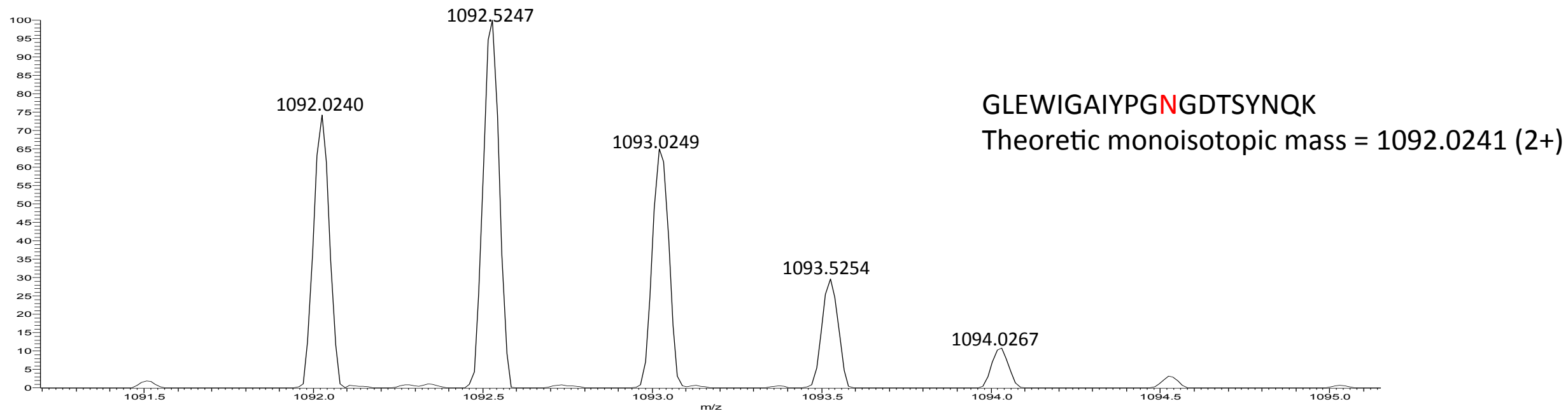

B.

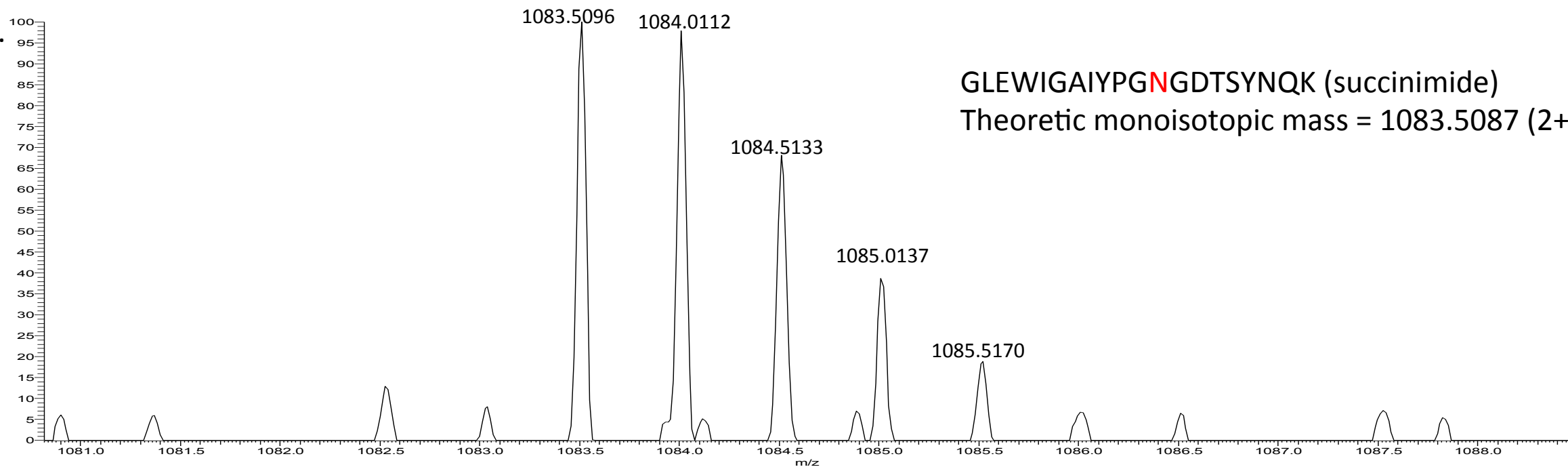

Supplement: Liu et al Supplemental Data [file kmab-08-02-1113361-s001.zip › SuppFigure2.pdf]

# Supplementary Figure 3

A.

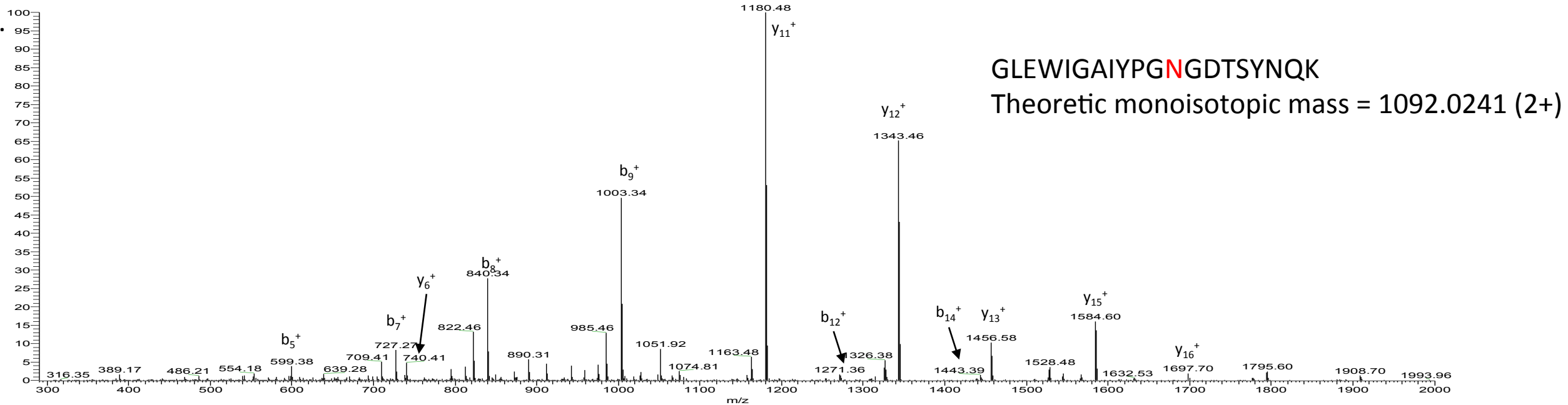

B.

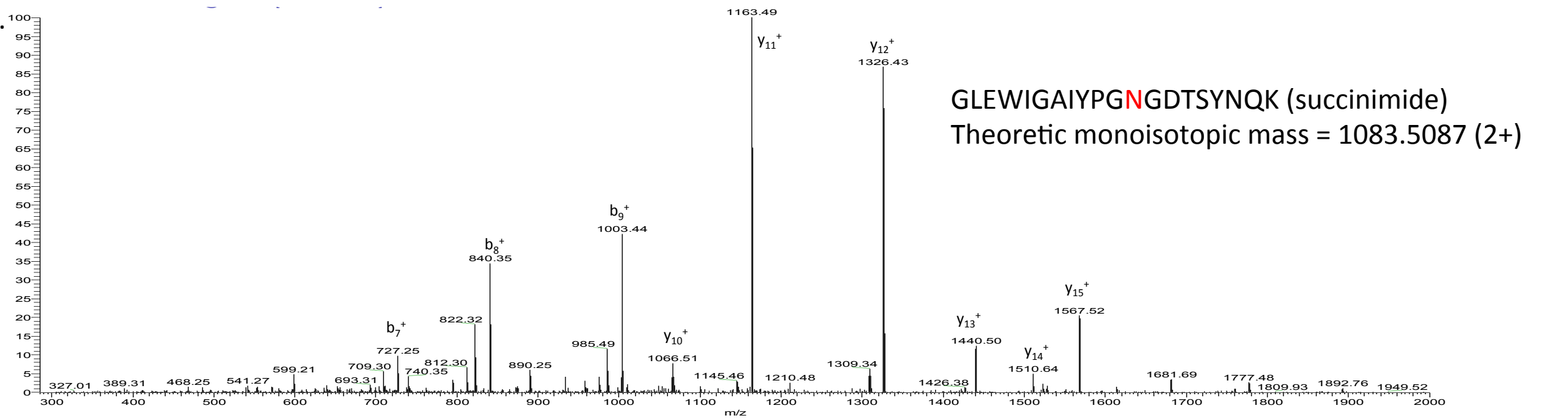

Supplement: Liu et al Supplemental Data [file kmab-08-02-1113361-s001.zip › SuppFigure3.pdf]

Supplementary Figure 4

A.

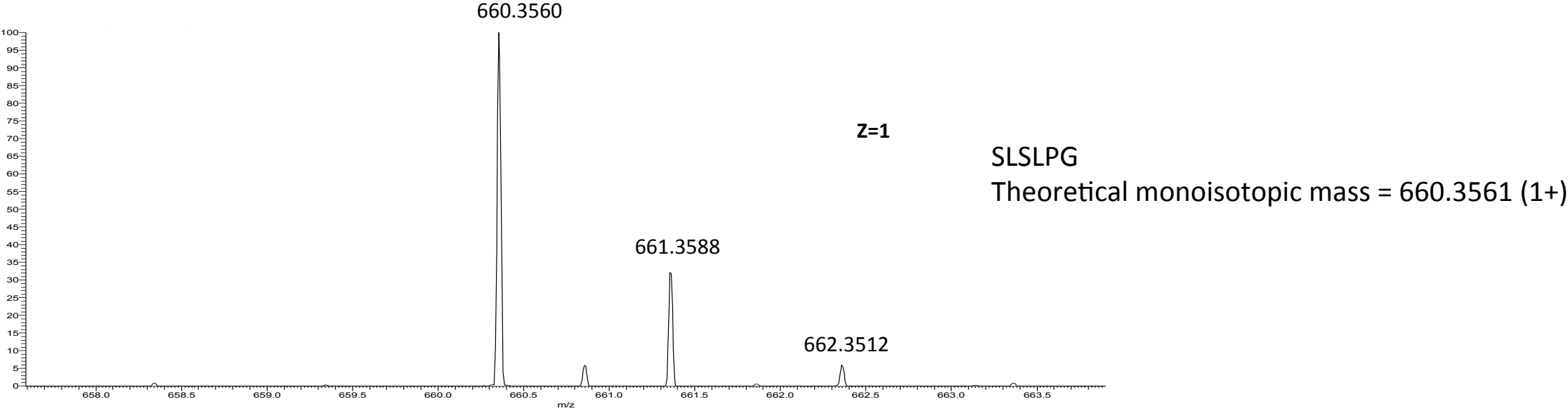

B.

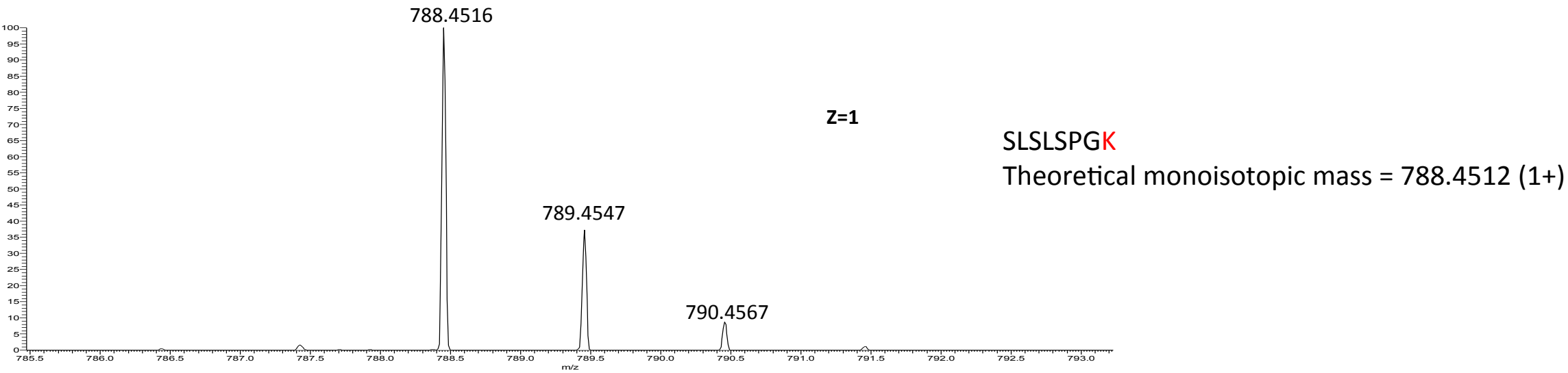

Supplement: Liu et al Supplemental Data [file kmab-08-02-1113361-s001.zip › SuppFigure4.pdf]

Supplementary Figure 5

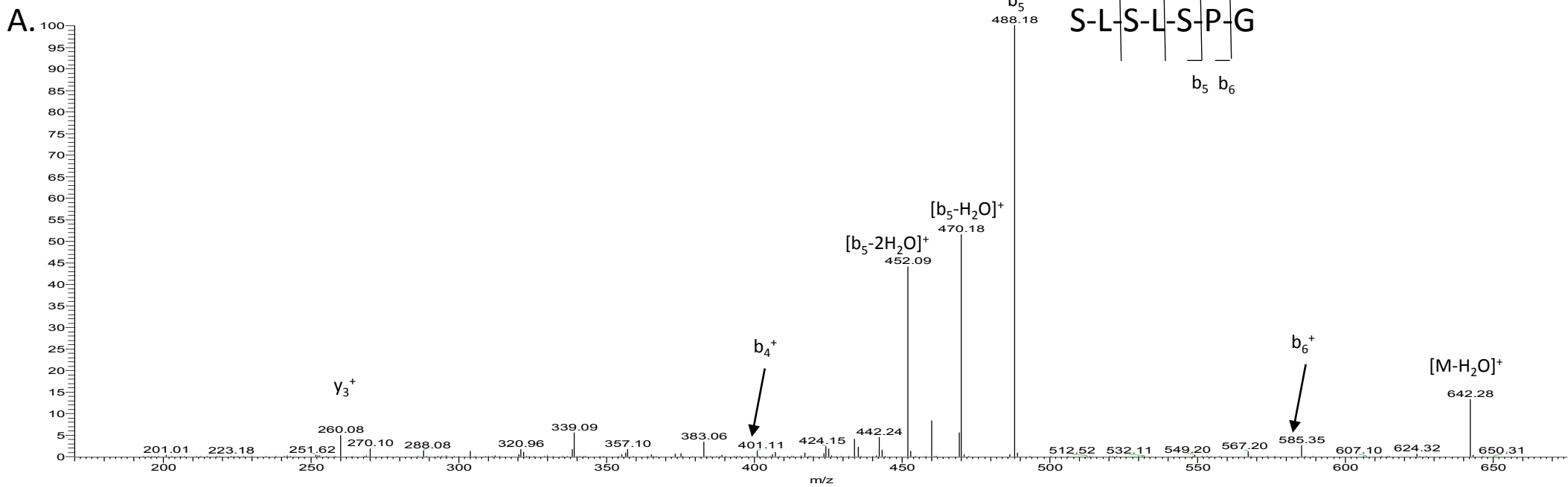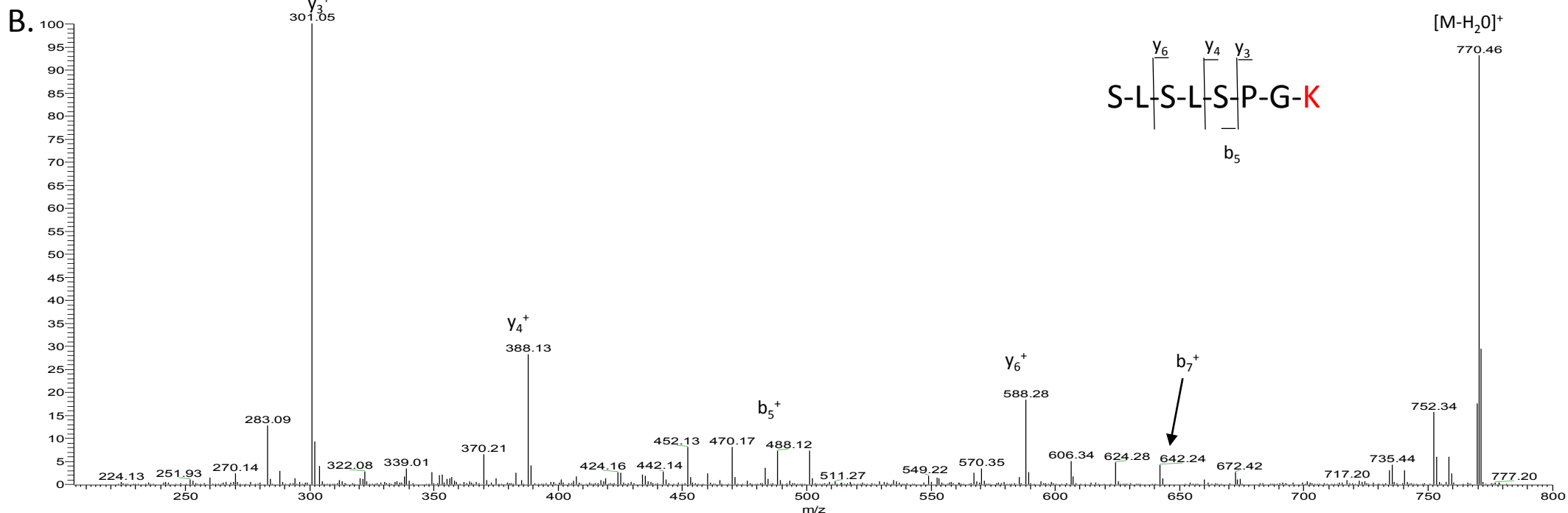

Supplement: Liu et al Supplemental Data [file kmab-08-02-1113361-s001.zip › SuppFigure5.pdf]

Supplementary Figure 6

A.

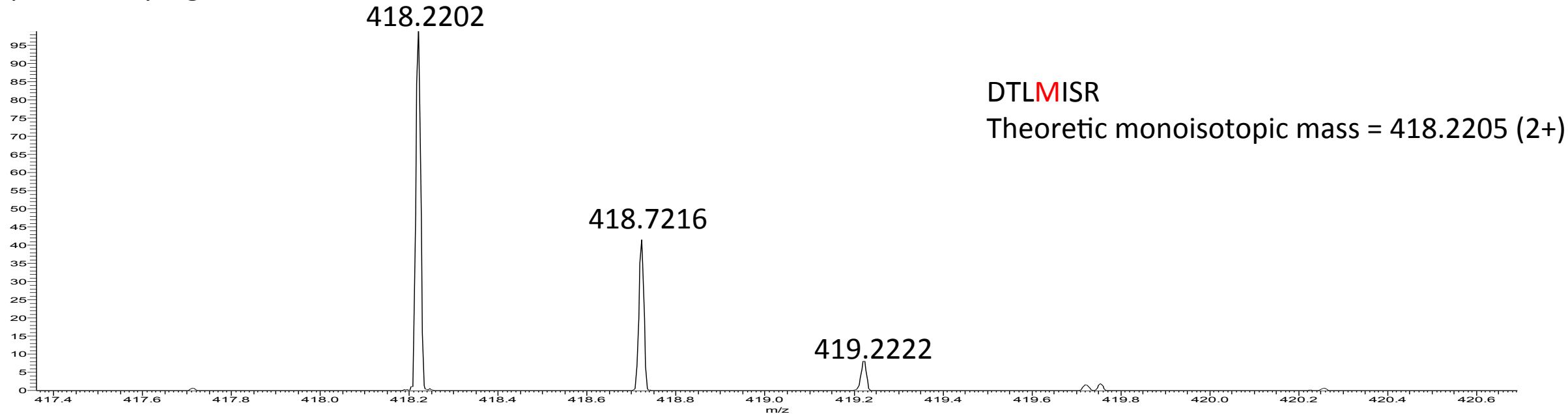

B.

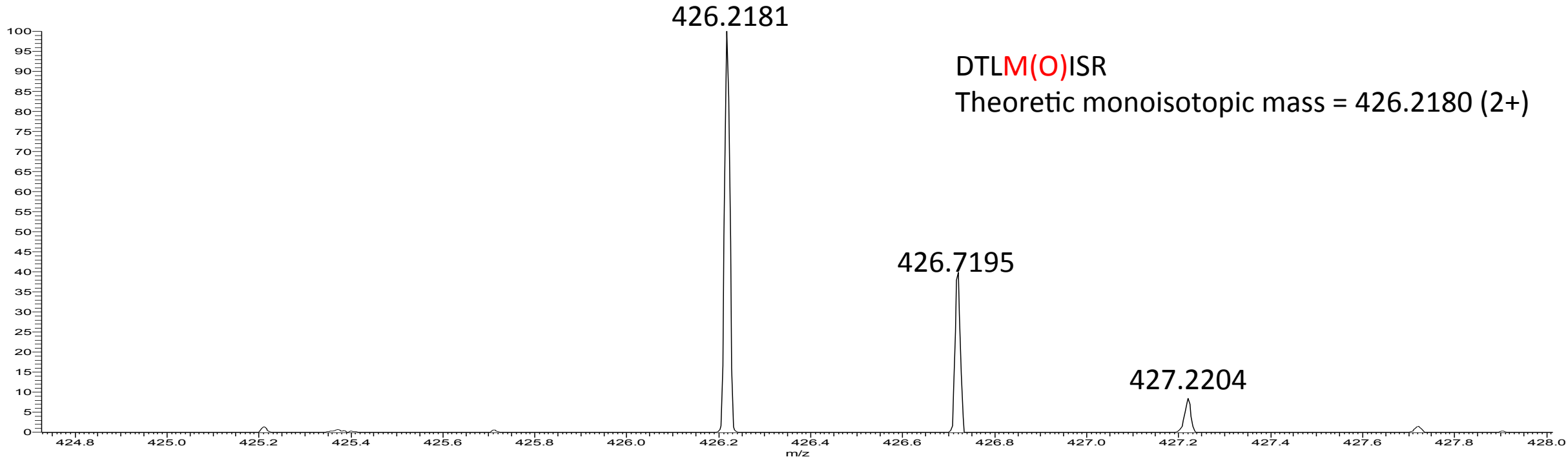

Supplement: Liu et al Supplemental Data [file kmab-08-02-1113361-s001.zip › SuppFigure6.pdf]

Supplementary Figure 7

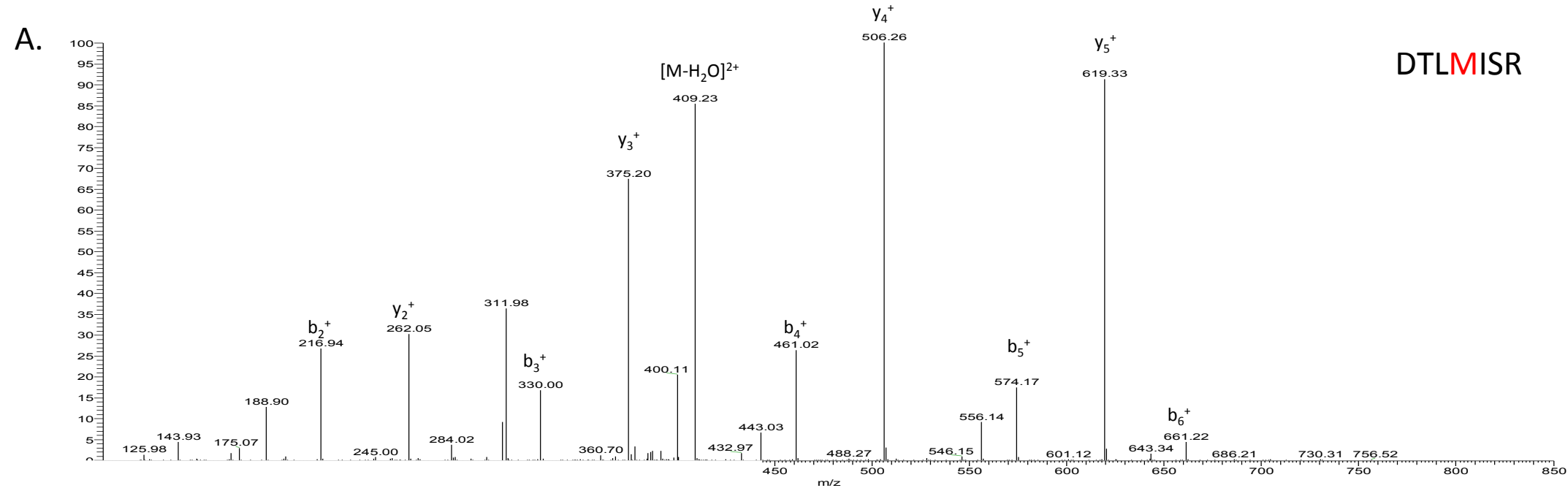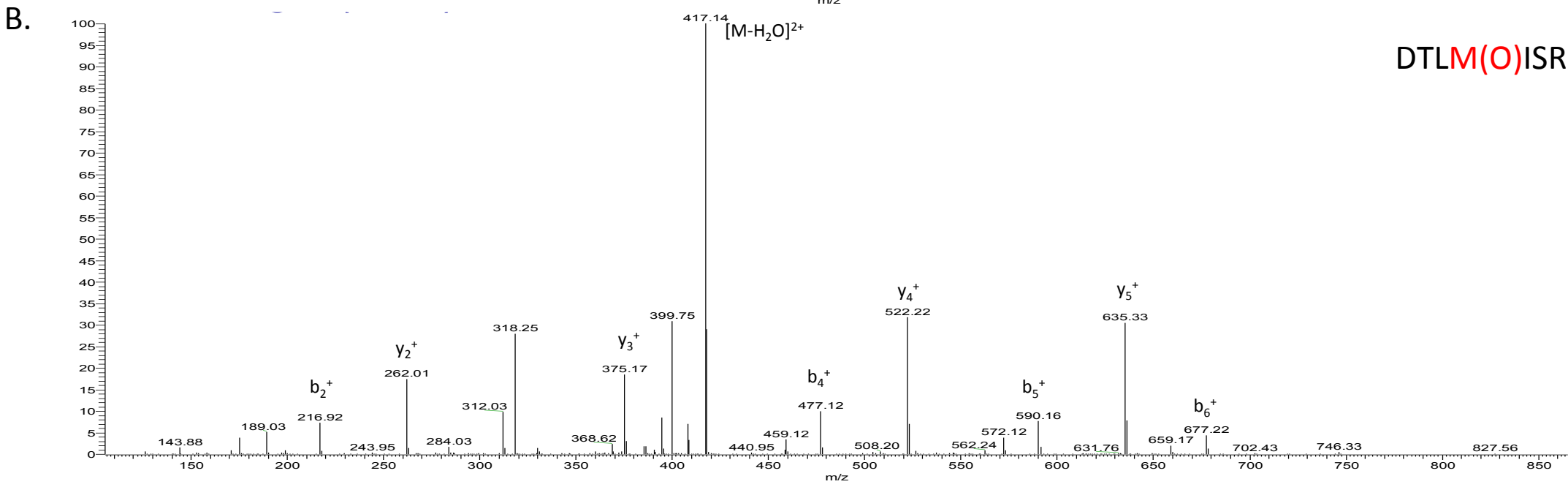

Supplement: Liu et al Supplemental Data [file kmab-08-02-1113361-s001.zip › SuppFigure7.pdf]
